# Database of the alumni performance of the Ophthalmology *Stricto sensu* Postgraduate Program at Escola Paulista de Medicina, UNIFESP^1^

**DOI:** 10.1590/s0102-865020200020000007

**Published:** 2020-04-17

**Authors:** Rosangela Demetrio, Rosely de Fátima Pellizzon, Flávio Eduardo Hirai, Denise de Freitas

**Affiliations:** I MD, Ophthalmology and Visual Science Department, Escola Paulista de Medicina (EPM), Universidade Federal de São Paulo (UNIFESP), Brazil. Conception, design, scientific and intellectual content of the study; acquisition, analysis and interpretation of data; statistics analysis; manuscript preparation and writing.; II MD, Library, EPM-UNIFESP, Sao Paulo-SP, Brazil. Critical revision.; III PhD, Ophthalmology and Visual Science Department, EPM-UNIFESP, Sao Paulo-SP, Brazil. Statistics analysis, critical revision.; IV PhD, Ophthalmology and Visual Science Department, EPM-UNIFESP, Sao Paulo-SP, Brazil. Scientific and intellectual content of the study, technical procedures, critical revision, final approval.

**Keywords:** Databases, Factual, Program Evaluation / standards, Education, Medical, Ophthalmology / education, Students, Medical

## Abstract

**Purpose:**

To develop a database with social, demographic and professional information of all graduates of the two post-graduate programs in Ophthalmology of EPM-UNIFESP, including their opinions on quality, application, and contribution of the courses received in their professional careers.

**Methods:**

The survey was conducted in the digital and physical archives of the University and by telephone contact. When the graduates’ e-mails were all collected, the electronic questionnaire was applied. The responses were compiled. Descriptive analysis of the results obtained in this cross-sectional study was performed, and analyzed by the authors and by statistical professionals, through Excel graphs.

**Results:**

The database suggests that most graduates were born and work in the state of São Paulo. A significant fraction of 66.77% is dedicated to academic work, but only 36.2% hold management positions. Most of them receive amounts of one to 56 minimum wages monthly. The main motivation was to improve their professional careers.

**Conclusion:**

For post-graduate programs, a database with information of its graduates can elucidate whether the goals were achieved based on the proposed teaching, as well as can generate reflections to improve the quality, the courses expectations and the vision that students have of the University.

## Introduction

Brazil has been developing great skills in research, science and technology^1^, and this takes more and more researchers with qualifications and experiences; accredited professionals who are enchanted by research and who intend to teach new generations the importance of knowing what a research is, following the research career. The permanence of a Postgraduate Program (PPG) at the maximum classification of grade 7 at *Coordenação de Aperfeiçoamento de Pessoal de Nível Superior (CAPES*), in addition to be directly linked to academic skills, may also be related to the actions and policies taken in terms of self-knowledge and the vision that the academic environment has of the Program.

The Ophthalmology and Visual Sciences PPG at Escola Paulista de Medicina (EPM) / Universidade Federal de São Paulo (UNIFESP) started in 1979 and, since April 2013, with the approval of the Professional Master course in Technology, Management and Eye Health by the Education Ministry (opinion MEC 271/2013)^2^, both programs have been training managers and professionals with practical qualification, based on the transfer of expertise, evidences, supplemented by teaching best practices in research laboratory and clinical analysis of scientific and technological management literature. In 38 years of existence, these PPG formed more than 199 masters with high qualification for teaching, 334 doctors and 15 post-doctors.

Evaluating the quality of training processes at all levels of education, especially masters and doctoral courses, and its relationship with the professional world, became part of the higher education institutions’ agenda. These assessments include the consequences of technological innovations, the demand for qualified researchers and the dissemination of a culture of evaluation^3^.

Based on this guideline, this study aimed to create a database profile of postgraduate students in order to provide an assessment with regard to the quality of the course and the fulfillment of the role of graduates in training human resources suitable for development research and teaching.

## Methods

The study participants are graduates of the Academic Master’s courses (174) and Professional Master (25) of Postgraduate in Ophthalmology and Visual Sciences and Technology, Management and Eye Health Programs of Escola Paulista de Medicina – Universidade Federal de São Paulo (UNIFESP), from 1979 to 2017. The research project was approved by the UNIFESP Research Ethics Committee (CAAE: 54180716.2.0000.5505 / Approval no. 1480825 / CEP-UNIFESP: 0224/2016).

For success in the search for data, it was necessary cooperation and permission of the Postgraduate Studies and Research Rectory of UNIFESP and the Management and Academic Secretary of the Ophthalmology and Visual Sciences Department, which provided agendas and spreadsheets. The data searches were also performed on the internet, in telephone directories, in the Lattes Platform and social networks. Several contacts were made by telephone in an attempt to get the e-mail addresses of graduates to date. Once in possession of these data, individuals were approached by e-mail and asked to fill a questionnaire.

The questionnaire consists of 45 questions, addressed from basic and demographic variables of each interviewee, such: as marital status, birth date, geographic location, undergraduate, postgraduate course, assistance from development agencies such as scholarship for pursuing studies, product of their study (thesis, dissertation, article published in a scientific journal, among other options), achievement motivation of the thesis, data about your current activity, such as current position occupying in the labor market and their income. Graduates also responded about the course’s faculty quality, adequacy of infrastructure and facilities, quality of equipment and if the postgraduate program helped in their training. The responses were compiled into a single database. Descriptive analysis of the results obtained in this cross-sectional study was performed, and analyzed by the authors and by statistical professionals, through Excel (Microsoft, Washington-WA, USA) graphs. Excel was used for tabulation and interpretation of research variables.

## Results

The population studied included 199 graduates who attended the Academic Master or the Professional Master in Ophthalmology and Visual Sciences programs and in Technology, Management and Eye Health. The sample achieved in this study comprised 36 individuals (18.09% of the participants) with a mean age of 46.2 years, ranging from 24.5 to 74.5 years. All respondents were Brazilian and most were married at the time of the survey (61.1%).

Most respondents were born in the state of Sao Paulo (77.77%), and the other 22.23% had their origin in the states of Bahia (2.77%), Federal District (2.77%), Minas Gerais (5.56%), Para (2.77%), Parana (2.77%), and Rio de Janeiro (2.77%). One of them did not answer (2.77%).

Regarding the question of the state in which they work, 32 subjects (88.9%) said that they work in the state of Sao Paulo. One works in the Federal District, one in the state of Minas Gerais, one in the state of Rio de Janeiro, and one did not answer (Chart 1).

**Chart 1:** Comparison of state of birth of individuals and state in which they work.

**STATE OF BIRTH**			**STATE OF WORK**				
		**Total**	**No reply**	**DF**	**MG**	**RJ**	**SP**
	**No reply**	1	- -	- -	- -	- -	1 100.0%
	**BA**	1	- -	- -	- -	- -	1 100.0%
	**DF**	1	- -	- -	- -	- -	1 100.0%
	**MG**	2	- -	- -	1 50.0%	- -	1 50.0%
	**PA**	1	- -	1 100.0%	- -	- -	- -
	**PR**	1	- -	- -	- -	- -	1 100.0%
	**RJ**	1	- -	- -	- -	1 100.0%	- -
	**SP**	28	1 3.6%	- -	- -	- -	27 96.4%
	**Total**	38	1 2.8%	1 2.8%	1 2.8%	1 2.8%	32 88.9%

When asked about the place in which they lived, 66.0% said they live in the state of Sao Paulo, 3.0% in Bahia, 3.0% in Rio de Janeiro, 3.0% in the Federal District and 25.0% did not answer this question.

At the time of this research, 67.0% of the sample (24 individuals) were employed in the education sector, 30.0% in the administrative sector, and 3.0% had no formal job. Among those 24 individuals linked to the education sector, 25.0% were working exclusively in the public sector, 25.0% only in the private sector, 8.33% only in a third sector institution (non-profit organizations), 8.33% had triple employment (public-private-third sector), 16.67% had double (public and private) employment, and 16.67% were working independently.

The gross monthly income of respondents (91.7%) ranges from one to 56 minimum wages. Only 8.3% reported receiving more than 56 minimum wages (a small group composed of individuals working only in the private sector). In the group of the sample who received between 6 and 10 minimum wages, 86.0% were in the public sector and 14.0% in the private sector. In the group of individuals with double employment (public-private), 50.0% received monthly, on average, 30 minimum wages; 25.0% received 14 minimum wages and 25.0% below this, which generated an average salary for the double-bonded individual of 18.75 monthly minimum wages. Individuals employed only in the public service received, on average, 7.85 minimum wages. Individuals working only in the private sector received an average monthly wage of 30.12 minimum wages. As a result, there was an important difference of 383.69% in income between individuals working in the private vs public sector only.

Regarding the studies conducted by the respondent individuals in the sample (91.7%), before the postgraduate course analyzed, 44.4% said they had attended a private educational institution, 33.4% had attended a Federal Public University, 8.3% had a State Public University and 5.6% had attended a local Public University.

The Professional Masters course included 67.0% of the sample and 33.0% the Academic Masters. The initial motivation for most postgraduate students (31.0%) was to acquire or improve knowledge, 25.0% were motivated by teaching, 25.0% for career progression at work, 11.0% for research and 8.0% did not answer. The final product for 22.0% of the students was a scientific publication, 64.0% did not have their thesis published, and 14.0% produced manuals and software.

All respondents confirmed that the course contributed to their vocational training. The results showed that for 69.4% of respondents, the postgraduate program cooperated to improve knowledge, 52.8% to career progression, 47.2% for research, 36.1% for teaching and 16.7% by status only.

When asked about the quality, 97.2% of subjects considered the faculty excellent or good and 91.7% considered the course an excellent or good learning opportunity. More details of the graduates’ opinion on the quality of the courses were also collected (Chart 2).

**Chart 2:** Evaluation of the quality of the course.

EVALUATION OF THE QUALITY OF THE COURSE							
	No reply	Excelent	Good	Medium	Regular	Terrible	Total
**QUALITY OF THE TEACHER**	- -	21 58.3%	14 38.9%	1 2.8%	- -	- -	36 100.0%
**CHALLENGING INTELLECTUAL ENVIRONMENT QUALITY**	- -	12 33.3%	17 47.2%	7 19.4%	- -	- -	36 100.0%
**QUALITY LEARNING OPPORTUNITY**		19 52.8%	14 38.9%	2 5.6%	1 2.8%	- -	36 100.0%
**QUALITY IN CURRICULUM ADEQUACY**	- -	9 25.0%	21 58.3%	4 11.1%	- -	2 5.6%	36 100.0%
**LIBRARY QUALITY ADEQUACY**	- -	6 16.7%	20 55.6%	5 13.9%	5 13.9%	- -	36 100.0%
**QUALITY IN INFRASTRUCTURE ADEQUACY**	- -	6 16.7%	16 44.4%	10 27.8%	2 5.6%	2 5.6%	36 100.0%
**EQUIPMENT QUALITY**	2 5.6%	9 25.0%	18 50.0%	4 11.1%	3 8.3%	- -	36 100.0%

## Discussion

Countries with high levels of social and economic development have, as a concern, the quality of postgraduate courses and the trends for choosing the research career^4^. To perform widely systematic follow-up studies of formation processes in the postgraduate program level in Brazil is to discuss successful experiences to build strategies for scientific development, that is, to identify the strengths and weaknesses of the courses to strengthen strict sense postgraduate programs^5^. The possibility of graduates evaluating their institution by the computerized system has been analyzed in the literature and it has been concluded that this assessment could reflect the quality and excellence of teaching^4^.

In the graduates population analyzed (199 individuals), only 18.09% (36) answered the questionnaire. A previous study^3^ has revealed that online questionnaires have low rates of acceptance by the respondents, but are increasingly used in cross-sectional studies, due to low cost and its possibility to reach different parts of the country and even abroad, being possible to quickly analyze the collected responses. Perhaps questionnaires should be shorter and simplified for easy filling, as well as should offer the possibility to be completed in several accesses.

Research conducted with the support of the Internet is very popular among researchers, mainly due to advantages such as: lower costs, speed and the ability to reach numerous and specific populations, ability to respond in the way that is most convenient at the time and place of each individual. On the other hand, there are drawbacks to consider, for example, low response rate to the questionnaires^7^. Anyway, there are more advantages than disadvantages in using the Internet for this type of satisfaction survey^9^.

The low response rate is the main drawback associated with the realization of an Internet marketing research^6^. Despite the disadvantages, the trend is that research with the use of questionnaires via email and electronic sites continue to occur, given the speed of expansion of Internet users^9^.

This preliminary study did not aim to present a broad overview of the graduates at the national level, as has been presented in the study entitled “Proposal for monitoring graduates of the Universidade Federal de Brasília based on a study of the follow-up of postgraduate programs nationwide^10^”. The purpose of this research was to implement a database to further detect points of difficulty and implement improvements in PPG courses. These data will allow to compose a future research, but this may be more comprehensive and complete with more consistent conclusions, with the largest population of graduates, as all postgraduate students of both programs will be analyzed.

The contact between the postgraduate program and its graduates was a positive outcome of this study. This research opened a channel of communication between the postgraduate program and its graduates. Increasingly, communication has been valued as a strategic instrument to add value to the image of any organization^11^.

The average age of individuals responding this study was 46.2 years, ranging from 24.5 to 74.5 years, while the age of the graduates of the Postgraduate Program in Medicine (Radiology), Faculdade de Medicina da Universidade Federal do Rio de Janeiro (UFRJ)^12^ ranged from 29 to 67 years, mean 43.5 years. By way of historical information, in 1980, when it was initiated the Postgraduate Program in Ophthalmology, Escola Paulista de Medicina-UNIFESP, five students did the course registration and all of them were male (100%). Today, according to data found, a total of 103 students are enrolled in both programs, including Master’s and doctoral academic, professional master’s and post-doctoral. Among these, 51 (49.5%) were female and 52 (50.5%) were male. The difference was only 1.0% between males and females enrolled in these postgraduate programs.

**Figure 1:**
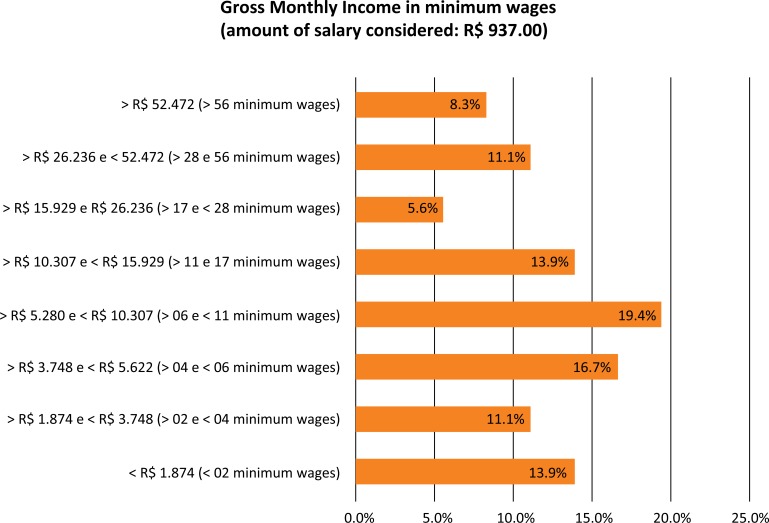
Gross monthly income in minimum wages (amount of salary considered: R$ 937.00).

Regarding the salary issue, this research revealed that the majority of respondent individuals, said they received a gross monthly income ranging between 6 and 11 minimum wages, as shown in Figure 1. According to the Data Analysis Research Graduates USP that investigated graduates of the Universidade de São Paulo (USP) in 2014, when the minimum wage was worth R$ 724.00^13^, t the majority (32.1%) of those interviewed said that their gross income was between R$ 6.000,00 (8.28 times the minimum wage in 2014) and R$ 10.000,00 (13.81 times the same minimum wage)^14^.

The biggest difference of the variables of income from one survey to the next, may be the fact that in the USP questionnaire, the range of greater wage value was as “more than R$ 15.000,00” (20.71 times the minimum wage 2014), while the UNIFESP fragmented a little more the salary ranges in the search, getting the most value searched in >R$ 52.472,00 or> 56 times the minimum wage (2017). When we take into account in this research the gross salary range of the USP research (those who receive over $ 15.000,00 (20.71 times the minimum wage in 2014), representing 10.5% of that survey respondents), we see that 25.0% of respondents are over R$ 15.929,00 (range that most closely matches the other search). Perhaps this wide variation in percentage of 10.5% (USP) to 25.0% (UNIFESP) in the item higher salary range is due to the fact that this research was carried out with the graduates of Ophthalmology, while the other carried out with the alumni of the Postgraduate Programs as a whole.

Regarding the graduation of the graduates, the research indicated a considerable difference of postgraduate students who attended private educational institutions for their bachelors with respect to those who attended public educational institutions. In this, 44.0% said they had graduated from private universities, 50.0% in public universities (federal, state, municipal and philanthropic) and 6.0% gave other answers or did not answer. In assessing the profile of graduates of the Postgraduate Program *stricto sensu* in Education of the Universidade Federal de Uberlândia (UFU)^15^, held from 2004 to 2009, 23.0% of subjects had a degree in private educational institutions, while 77.0% at public institutions (federal, state, municipal and philanthropic)^15^. We can notice a much more subtle difference between the graduates of UNIFESP (44.0% private to 50.0% public), compared to the graduates of UFU (23.0% private to 77.0% public).

The Graduates Monitoring Program of Civil Engineering in 2013, made by the Faculdade de Engenharia São Paulo (FESP)^16^, reveals that “from respondents who were enrolled in postgraduate courses, 41.0% (most) said they studied to deepen knowledge, 22.0% by the market requirement, 8.0% for an academic career, 8.0% for promoting employment, 4.0% for other reasons unlisted and 16.0% did not answer. As for the initial motivation for attending postgraduate course, this research reveals that the majority (30.6%) sought to acquire or improve knowledge, 25.0% were motivated by teaching, 25.0% did the postgraduate program to career progression at work and 11.1% for the research. What is striking in the comparison of data between the motivation of graduates in Ophthalmology and Visual Sciences Program of Escola Paulista de Medicina / UNIFESP and the postgraduate of the Faculdade de Engenharia São Paulo (FESP), is perhaps the choice of career of teaching. In this survey, 25.0% of subjects responded that the motivation was to follow teaching, i.e., aimed to become teachers in the segment. While in the survey by FESP^16^, only 8.0% answered that their motivation was an academic career.

In the profile analysis of the graduates of the Postgraduate Program in Medicine (Radiology), Faculdade de Medicina da Universidade Federal do Rio de Janeiro^12^, 39.07% of respondents said their motivation was improvement of technical and scientific skills, 26.82% were aimed at teaching progression, 19.16% attended postgraduate school for personal satisfaction, 8.05% to 6.90% to recycle knowledge and to gain a better pay. The 26.82% aimed at teaching denotes an approximate proportion of this research; it was 25.0%. It is possible that medical courses are most valued by future graduates and promote more interest in teaching compared to the course of Engineering.

It is worth mentioning here that the search for the teaching profession is also an improvement of knowledge, as it means the person wants to be respected, improving his or her wage and living conditions more broadly. And this leads us to values inherent in the family background of each individual. When parents did not have opportunities for intellectual and professional growth, they fight for their children to attend the best schools, seeking to value and perform their professional activity^15^.

The percentage of 50.0% of individuals who claimed to have ties in the public work sector does not come as a surprise in this research because as a student of a public education institution, the student has the opportunity to experience the routine of professors and researchers. When they go to the labor market, certainly they begin to compare behaviors and ideologies. In addition to the personal issues that may exist, they also value the stability of public servants, which is a right guaranteed by the 1988 Federal Constitution^17^.

Regarding the study “Postgraduate Program Graduates profile evaluation in the *stricto sensu* of Education of the Universidade Federal de Uberlândia: impact of teacher education and research”, it is observed that most of the interviewees worked as a teacher in public universities, being employed in the public sector^15^. According to the same study data, the graduates had a career in public service, improved their training as teachers and researchers, to later return to the same type of employment. These data are consistent with Velloso^18^, who, in his study, found that almost 85.0% of the participants of his research worked in university and/or research institutions.

Although the study population was restricted to one area of Medicine, Ophthalmology, it can be concluded that the results can contribute continuously to the curriculum formulation by the course coordinators and support the improvement of academic activities and guidelines, based on the satisfaction and expectation of the graduates. It might be useful as a warning so that more attention is focused on the monitoring of graduates, it is based on the analysis of their profile, their achievements and trajectories that postgraduate quality may be evaluated.

Another aspect of the research is that it will provide the basis for future studies, comprising a large population of respondents, considering that a second phase of the study is expected, addressing all individuals trained by the PPG in Ophthalmology and Visual Sciences and Technology, Management and Eye Health, both master, doctoral and post-doctoral.

## Conclusion

For post-graduate programs, a database with information of its graduates can elucidate whether the goals were achieved based on the proposed teaching, as well as can generate reflections to improve the quality, the courses expectations and the vision that students have of the University.

## Footnotes

1 Research performed at Ophthalmology and Visual Science Department, Escola Paulista de Medicina (EPM), Universidade Federal de Sao Paulo (UNIFESP), Brazil.
